# Plasmacytoid Dendritic Cells Provide Protection Against Bacterial-Induced Colitis

**DOI:** 10.3389/fimmu.2019.00608

**Published:** 2019-04-09

**Authors:** Tania Rahman, Andrew S. Brown, Elizabeth L. Hartland, Ian R. van Driel, Ka Yee Fung

**Affiliations:** ^1^Department of Microbiology and Immunology, University of Melbourne at the Peter Doherty Institute for Infection and Immunity, Melbourne, VIC, Australia; ^2^Department of Biochemistry and Molecular Biology, Bio21 Molecular Science and Biotechnology Institute, University of Melbourne, Melbourne, VIC, Australia

**Keywords:** plasmacytoid dendritic cells, *Citrobacter rodentium*, bacterial gut pathogen, colitis, cytokines

## Abstract

We have examined the influence of depleting plasmacytoid dendritic cells (pDC) in mice on the immune response to the gut pathogen *Citrobacter rodentium*, an organism that is a model for human attaching effacing pathogens such as enterohaemorraghic *E. coli*. A significantly higher number of *C. rodentium* were found in mice depleted of pDC from 7 days after infection and pDC depleted mice showed increased gut pathology and higher levels of mRNA encoding inflammatory cytokines in the colon upon infection. pDC-depletion led to a compromising of the gut mucosal barrier that may have contributed to increased numbers of *C. rodentium* in systemic organs. pDC-depleted mice infected with *C. rodentium* suffered substantial weight loss necessitating euthanasia. A number of observations suggested that this was not simply the result of dysregulation of immunity in the colon as pDC-depleted mice infected intravenously with *C. rodentium* also exhibited exacerbated weight loss, arguing that pDC influence systemic immune responses. Overall, these data indicate that pDC contribute at multiple levels to immunity to *C. rodentium* including control of bacterial numbers in the colon, maintenance of colon barrier function and regulation of immune responses to disseminated bacteria.

## Introduction

Plasmacytoid dendritic cells (pDC) are developmentally and functionally distinct from antigen-presenting, conventional DC (cDC) (1). One of the important roles for pDC is linking innate immunity to adaptive immunity. They are well-known for secreting large amounts of type I interferons (IFNs) in response to viral infection (2); pDCs produce 10–100-fold more type I IFN than other immune cells following their recognition of unmethylated CpG dinucleotides and double-stranded RNA synthesized by many viruses through toll like receptor 7 (TLR7) and TLR9, respectively (3). Apart from type I IFNs, pDCs also produce pro-inflammatory cytokines such as interleukin (IL)-6 (IL-6), IL-12, and tumor necrosis factor (TNF) in response to TLR activation, thus activating the adaptive immune response (4, 5).

In contrast to their well-established immunoregulatory role during viral infection, little is known about the role of pDCs in bacterial infection. Several studies have shown that pDCs are activated by *Citrobacter rodentium* (6), *Borrelia burgdorferi* (7), *Toxoplasma gondii* (8), and *Staphylococcus aureus* (9) and produce cytokines including type I IFN and IL-12. Moreover, a recent report showed that mouse pDC express TLR2 which detects polysaccharide A, an immunomodulatory molecule expressed by the gut microbiota *Bacteroides fragilis* and induces IL-10 production by T regulatory cells (10).

*Citrobacter rodentium* is a Gram negative natural mouse pathogen that is closely related to the attaching and effacing human pathogens, enteropathogenic and enterohemorrhagic *E. coli* (EPEC/EHEC). These pathogens attach intimately to enterocytes and induce damage to the mucosal barrier. Both innate and adaptive immune responses are required to provide optimal protection against *C. rodentium*. For example, IL-22 producing Group 3 innate lymphoid cells (11) and IL-17 producing CD4^+^ T cells (12) are essential for protection. However, the role for pDC and type I IFNs in gut bacterial infection generally, and *C. rodentium* infection in particular, has not been documented.

In the present study, we investigated the *in vivo* role of pDC in the immune response to *C. rodentium*. We demonstrated that pDC infiltrate the colon after *C. rodentium* infection and that pDC-depleted mice were highly susceptible to *C. rodentium* infection, exhibiting increased bacterial numbers in fecal samples, increased systemic dissemination, and systemic inflammation. Based on our findings, we propose that pDC are required for optimal protection in both gut mucosal and systemic immunity during *C. rodentium* infection.

## Materials and Methods

### Antibodies and Reagents

The following antibodies were purchased from eBioscience: Pacific Blue conjugated anti-mouse CD4 (clone: RM4-5), FITC conjugated anti-mouse CD45.1 (clone: A20), biotin conjugated anti-mouse CD3 (clone: 17A2), FITC conjugated anti-mouse CD3 (clone: 145-2C11), APC conjugated anti-mouse CD317 (BST2, PDCA1) (clone: eBio927), Pacific Blue conjugated anti-mouse CD11c (clone: N418), FITC conjugated anti-mouse MHCII (I-A) (clone: NIMR-4), biotin conjugated CD317 (BST2, PDCA1) (clone: eBio927), PerCP-Cy5.5 conjugated anti-mouse Ly-6G (Gr-1) (clone: RB6-8C5), streptavidin conjugated PerCP-Cy5.5. The following antibodies were from BD Pharmingen: PE-Cy7 conjugated anti-mouse CD45 (clone: 30-F11), PerCP conjugated anti-mouse CD4 (clone: RM4-5), PE conjugated anti-mouse CD19 (clone: 1D3), APC-Cy7 conjugated anti-mouse CD11b (clone: M1/70), Biotin conjugated anti-mouse Ly-6C (clone: AL-21), PerCP-Cy5.5 conjugated anti-mouse CD45R (B220) (clone: RA3-6B2), PE conjugated anti-mouse Ly-6G (clone: 1A8), PerCP-Cy5.5 conjugated Ly-6C (clone: AL-21), streptavidin conjugated V500. Diphtheria toxin (DT, isolated from *Corynebacterium diphtheriae*) was purchased from Sigma-Aldrich (St Louis, MO). Mouse Th1/Th2/Th17 Cytokine kit (BD Biosciences, California, USA) was obtained from BD^TM^ Cytometric Bead Array (CBA).

### Mouse Strains, Bacterial Strains, and Methods

All animal experiments were approved by the University of Melbourne Animal Ethics Committee (ethics approval number 1413406.5). Wildtype C57BL/6 mice, C57BL/6-Tg(CLEC4C-HBEGF)^956Cln^ (BDCA2-DTR) mice (13), B6(Cg)-*Ifnar1*^*tm*1.2*Ees*^ (IFNAR1^−/−^) (14), and *Ptprc*^*a*^ (CD45.1) mice were bred and housed under specific pathogen free conditions at animal facilities at the University of Melbourne.

A spontaneous nalidixic acid (Nal) resistant derivative of *C. rodentium* biotype 4280 (ICC169), obtained from G. Frankel (Imperial college, London) was used in this study. Bacterial inocula were grown in Luria-Bertani (LB) broth containing 50 μg/mL Nal, overnight at 37°C with shaking. On the following day, mice were infected. Two infection routes were used. Firstly, oral gavage with 1 × 10^9^ CFU in PBS. Secondly, i.v. in which case mice were injected with 1 × 10^6^ CFU on days 0, 2, 4, 6, and 8. Mice were weighed and monitored daily after inoculation.

*In vivo* depletion of pDC was performed by injecting DT (300 ng/mouse) into the peritoneal cavity in a total volume of 200 μL per mouse on the day before (day -1) infection with *C. rodentium* on day 0. After infection, mice received 5 additional 300 ng doses every second day. Control groups were injected i.p. with equivalent volumes of sterile PBS.

To assess bacterial colonization, fecal pellets were collected aseptically from each mouse every alternate day and emulsified in PBS at a final concentration of 100 mg/mL. The number of viable bacteria per gram of feces was determined by plating serial dilutions of the samples onto LB plates containing Nal, as above. To assess bacterial colonization in spleen or liver, organs were weighed and homogenized in PBS before plating as above.

The extent of *in vivo* intestinal permeability was determined using 4 kDa FITC–dextran (Sigma-Aldrich) in serum. Briefly, mice were fasted for 5 h and were administered with 12 mg FITC-dextran in PBS by oral gavage. Serum samples were obtained 4 h later by cardiac puncture and FITC levels were determined using a fluorescence spectrometer (excitation, 488 nm; emission, 530 nm).

### Flow Cytometry

Colons were cut in to small pieces (approximately 2 mm^3^) and washed with PBS. Colon tissues were then digested with 3 mL digestion media containing RPMI-1640 media (GIBCO Thermo Fisher Scientific, USA) with 10 % FCS (v/v), 0.1 % (w/v) DNAse (Sigma-Aldrich, Missouri, USA) and 0.1 % (w/v) Collagenase type III (Washington Biochemical Corporation, USA) and incubated at 37°C for 45 min. Undigested colonic pieces were then filtered through a nylon mesh column in a 10 mL syringe to produce single cell suspensions. Spleens and lymph nodes were digested with digestion media (see above). pDC were enriched by Nycodenz medium (Nycomed, Oslo, Norway) as previously described (15). Undigested material was filtered with 70 μm filters (Corning) to produce single cell suspensions. Single cells were stained using antibodies described above. Total numbers for pDC were enumerated from the tissues by addition of a known quantity of APC-labeled microspheres (BD Calibrite) to each sample prior to acquisition in a LSRFortessa™ flow cytometer (BD). Data was analyzed using FlowJo software (FlowJo, LLC).

### Histopathological Analysis

For histological analysis, mice were killed at day 10 and whole colons were collected and fixed in 4% (w/v) paraformaldehyde (Sigma-Aldrich) and sectioned for hematoxylin and eosin staining for assessment of gut pathology by a pathologist at the Australian Phenomics Network. A scoring system (0–3) was used by a veterinary pathologist to assess the extent of degrees of change. Crypt Architecture: 0 = normal, 1 = irregular, 2 = moderate crypt loss, 3 = severe crypt loss; Tissue damage: 0 = no damage, 1 = discrete lesion, 2 = mucosal erosion, 3 = extensive mucosal damage/ulceration (extending into muscularis and deeper); Inflammation: 0 = occasional infiltration, 1 = increasing leukocyte in lamina propria, 2 = confluence of leukocytes extending into submucosa, 3 = transmural extension of inflammatory infiltrate; Enterocyte hyperplasia: 0 = none, 1 = mild, 2 = moderate, 3 = severe.

### pDC Reconstitution

To generate large quantities of pDC, C57BL/6 mice were subcutaneously injected with 5 × 10^6^ B16-Flt3L melanoma cells. After 11–13 days, pDCs were isolated and enriched from spleen as described above. Flow cytometry-purified pDC cells (~10^5^ per mouse) were transferred to recipient BDCA2-DTR mice by intravenous route on day 1, 3, 5, 8 after infection.

### Measurement of Cytokine Levels in Mouse Serum

Mouse serum was obtained by cardiac puncture and was used to analyse cytokines and chemokines by BD Cytometric Bead array Mouse Th1/Th2/Th17 Cytokine kit (BD Biosciences, California, USA) as per the manufacturer's instructions.

### Quantitative Reverse Transcriptase Polymerase Chain Reaction

Distal colon (~0.5 cm) was collected into RNAlater (Sigma) and homogenized in TRIsure TRI-reagent (Bioline). mRNA was extracted and cDNA was synthesized as previously described (16). Primers for genes products are described in Supplementary Table 1 and were used in conjunction with SSOAdvanced Universal SYBR Green Supermix (Biorad) to quantitate relative levels of these genes. qRT-PCR analyses was performed using a Quantstudio 7 Flex Real Time PCR System (Applied Biosystems).

### Statistical Analysis

Statistical analysis of the results was calculated using non-parametric two-tailed Mann-Whitney *U*-test. A *P*-value of <0.05 was the criterion for a statistically significant difference. Analyses were performed using GraphPad Prism 5 (Graph Pad Software, Inc., California, USA).

## Results

### Mice Depleted of pDC Exhibited Increased Weight Loss, Bacterial Load and Mucosal Pathology After *C. rodentium* Infection

**Figure 1:**
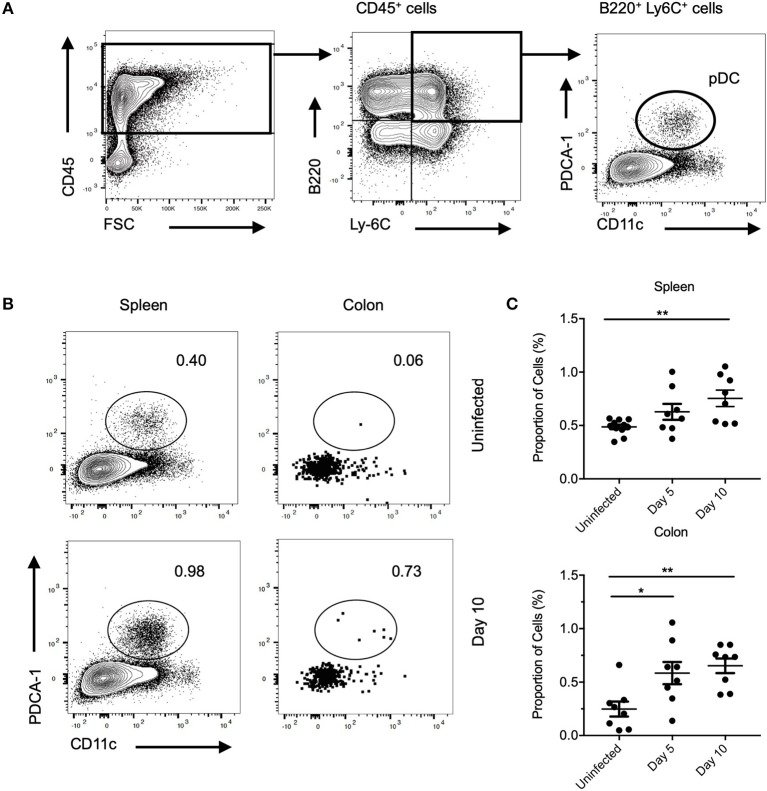
pDC in mice during *C. rodentium* infection. **(A)** Flow cytometric gating to identify pDC in cells isolated from organs of mice. Example shown is from spleen. pDC were identified as the PDCA-1^+^CD11c^low^ cells in the B220^+^Ly-6C^+^CD45^+^ population. **(B)** Representative flow cytometric plots from spleen and colon from uninfected mice and mice 10 days after *C. rodentium* infection (Day 10). **(C)** Proportion of CD45^+^ cells identified as pDC in uninfected mouse spleen and colon or after *C. rodentium* infection at the days indicated. Each dot represents data for samples from one mouse. Mann-Whitney *U*-test, ^*^P < 0.05, ^**^P < *0.01*.

To investigate the role of pDC in gut mucosal bacterial infection, we first examined pDC recruitment to colons of mice infected with *C. rodentium*. C57BL/6 mice were infected with *C. rodentium* for 5 or 10 days and pDC infiltration was analyzed by multi-color flow cytometry. The gating strategy used to identify pDC is shown in Figure 1A and representative plots of pDC in the spleen and colon at day 10 after infection is shown in Figure 1B. At day 5 and 10 after infection, pDC constituted a moderately increased percentage of CD45^+^ cells in the colon compared to steady-state Figure 1C although the number of pDC in these organs was not significantly increased (Supplementary Figures 1A,B).

**Figure 2:**
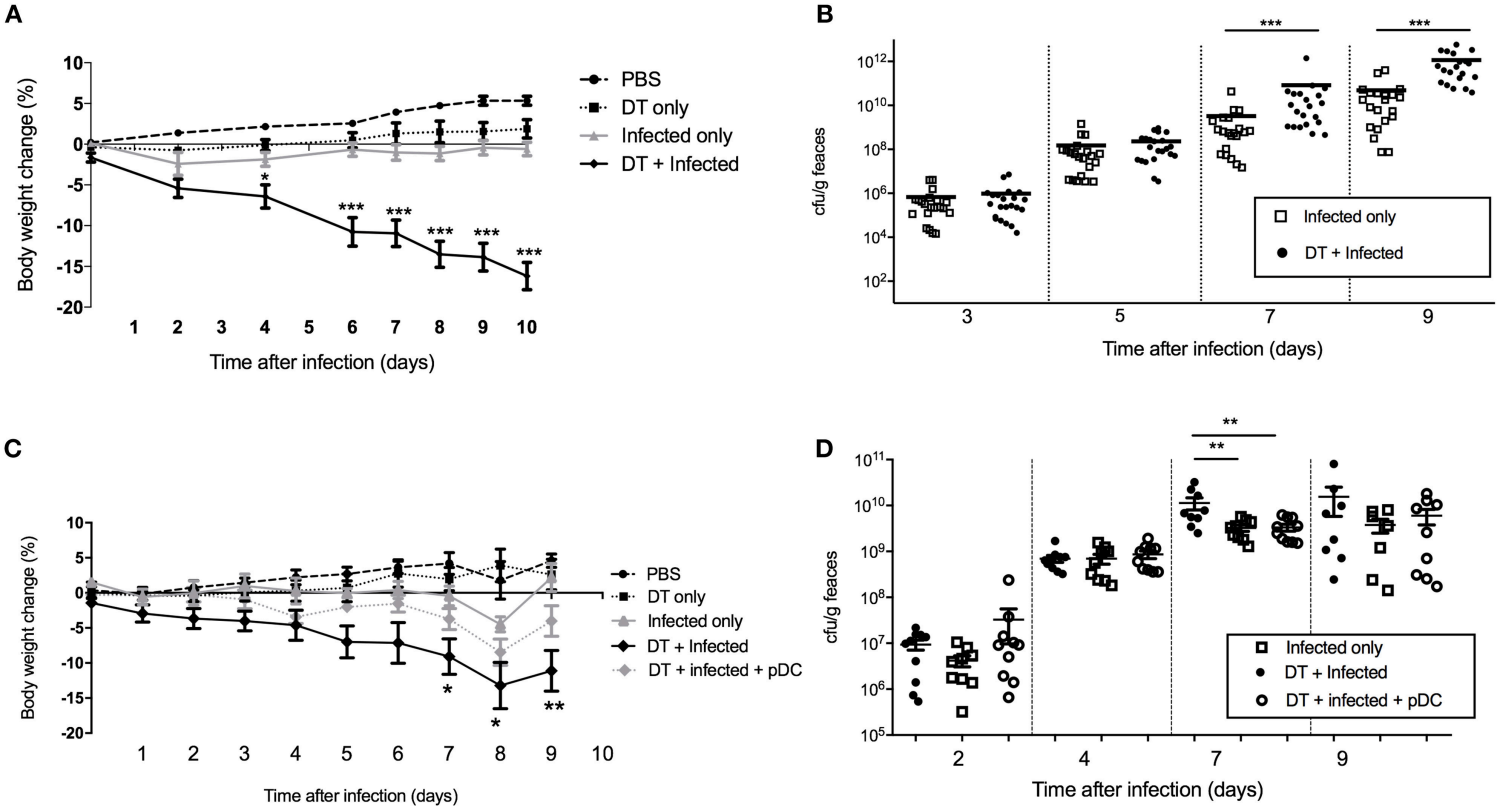
Mice deficient in pDC are more susceptible to *C. rodentium* infection. BDCA2-DTR mice were treated as indicated. **(A)** Weight change of mice during the course of infection. Data expressed as mean ± s.e.m., *n* = 5 per group in three independent experiments. **(B)** *C. rodentium* CFU in fecal pellets. Each symbol is data for one mouse. For **(C,D)** BDCA2-DTR mice were treated was PBS (PBS and “Infected only”), DT (“DT only”, “DT + Infected” and “DT + Infected + pDC”) 1 day prior to oral *C. rodentium* infection. pDC injected into “DT + Infected + pDC” mice on day 1, 3, 5, and 8 days after *C. rodentium* infection. **(C)** Weight change of mice during the course of infection. Asterisks indicate *P*-values between the DT + Infected and DT + Infected + pDC groups **(D)** *C. rodentium* CFU in fecal pellets. Each symbol is data for one mouse. Mann-Whitney *U*-test, ^*^P < 0.05, ^**^P < 0.01, ^***^P < 0.005.

In order to study the role of pDC in protection against infection, BDCA2-DTR mice (13) in which pDC are depleted upon exposure to DT were challenged orally with *C. rodentium*. pDC were ablated by DT injection on the day before *C. rodentium* infection (day -1) and subsequently on days 1, 3, 5, 7, 9 after infection. On average, we found ~90% of pDC were depleted in the spleen and ~50% in the colon (Supplementary Figures 1C,D). There was no depletion of neutrophils, macrophages or monocyte-derived cells in DT treated mice relative to the appropriate controls. We found that infected mice exposed to DT (“DT + Infected”) began to lose weight 2 days after infection and by day 10 had lost more than 15% of their body weight on average (Figure 2A). These mice had to be killed due to their weight loss approaching an ethically unacceptable limit. On the other hand, the weight of the mice not treated with DT and *C. rodentium*-infected (“Infected only”) varied only slightly during the course of infection (Figure 2A). The weight loss of “DT + Infected” group was not a side-effect of DT treatment because there was no weight loss in the group that received DT only (Figure 2A, “DT only”). In addition, we determined the number of viable *C. rodentium* in fecal samples (Figure 2B). The “DT + Infected” group had significantly higher bacterial loads than non-depleted, “Infected only” mice on days 7 (~10-fold) and 9 after infection (~100-fold).

To confirm that the increased weight loss and increased bacterial load observed in the BDCA2-DTR mice treated with DT was due to pDC depletion, we reconstituted pDC-depleted, *C. rodentium* infected mice with 1 × 10^5^ purified pDC (“DT + Infected + pDC,” Figure 2C, Depletion and reconstitution shown in Supplementary Figure 1E). Unlike the “DT + Infected” group in this experiment, “DT + Infected” mice that received pDC had similar weight loss to non-pDC-depleted mice that were infected. Furthermore, DT-treated mice that were infected and reconstituted with pDC did not have significantly different fecal bacterial loads compared to the “Infected only” group (Figure 2D). Both of these groups had lower bacterial loads than the “DT + Infected” group at day 7 after infection.

**Figure 3:**
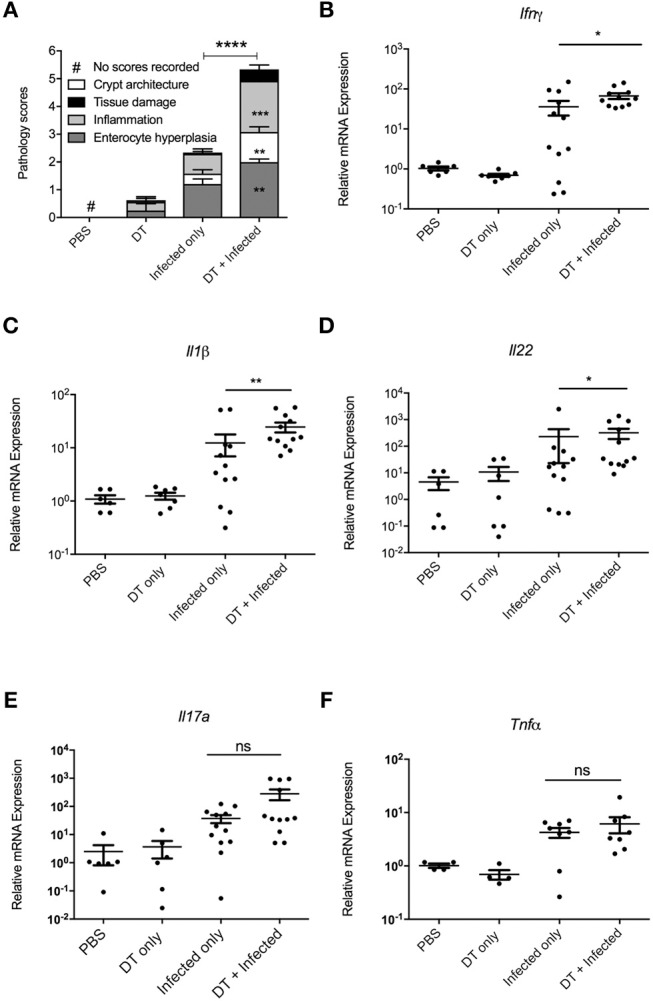
pDC deficiency leads to increase in histopathological scores and cytokine production in colons of *C. rodentium* infected mice. BDCA-DTR mice treated as indicated were analyzed 10 days after infection. **(A)** Histopathological analysis of the characteristics indicated. Data expressed as mean ± s.e.m., *n* = 5 per group in three independent experiments. **(B–F)** mRNA levels of the indicated cytokines as determined by quantitative PCR. Mann-Whitney *U*-test, ^*^P < 0.05, ^**^P < 0.01, ^***^P < 0.005, ^****^P < 0.0001.

In order to examine whether the elevated bacterial load in “DT + Infected” mice correlated to an increase in colon tissue pathology, the histopathological presentation of stained colon sections was examined on day 10 after infection (Figure 3A, Representative images are shown in Supplementary Figure 2). Histopathology was examined and scored for tissue damage, changes in crypt architecture, inflammation and enterocyte hyperplasia. “DT + Infected” mice had significantly higher pathological scores for abnormal crypt architecture with loose contents but intact mucosa, increased inflammation of the muscularis, submucosa characterized by neutrophils and lymphocytes and moderate to severe enterocyte hyperplasia and overall pathological scores when compared to “Infected only” mice. Tissue damage scores were not significantly different between the two groups of mice. Also, moderate bacterial colonization within crypts and mucosal surface were found in “DT + Infected” mice but not in “Infected only” mice.

The increase in bacterial load and inflammation suggested that there would be increased inflammatory gene expression in colon tissue. Therefore, we investigated levels of mRNA expression of genes encoding inflammatory cytokines *Ifn-*γ (17), *Il-1*β (18), *Il-22* (11), *Il-17a* (12), and *Tnf*α (19), which have been shown to play a critical role in protection against *C. rodentium* infection. As shown in Figures 3B–F, expression of *Ifn-*γ, *Il-1*β, and *Il-22* was significantly higher in the colon of “DT + Infected” group compared to “Infected only” mice, although gene expression of *Il-17a* and *Tnf*α was increased to a similar level between the two groups.

We have investigated the number and proportion of CD4 T cells, Treg cells and B cells in mesenteric lymph node and colon (Supplementary Figure 3) and we found no differences between the groups of mice that were infected compared to those infected and depleted of pDC.

Overall, pDC depletion converted *C. rodentium* infection from a benign resolving condition with low levels of colonic inflammatory damage and moderate influence on overall health into severe colitis with significant weight loss necessitating euthanasia.

### pDC Depletion Results in Increased Intestinal Permeability, Bacterial Dissemination and an Elevation of Inflammatory Cytokine Levels in Serum of *C. rodentium* Infected Mice

**Figure 4:**
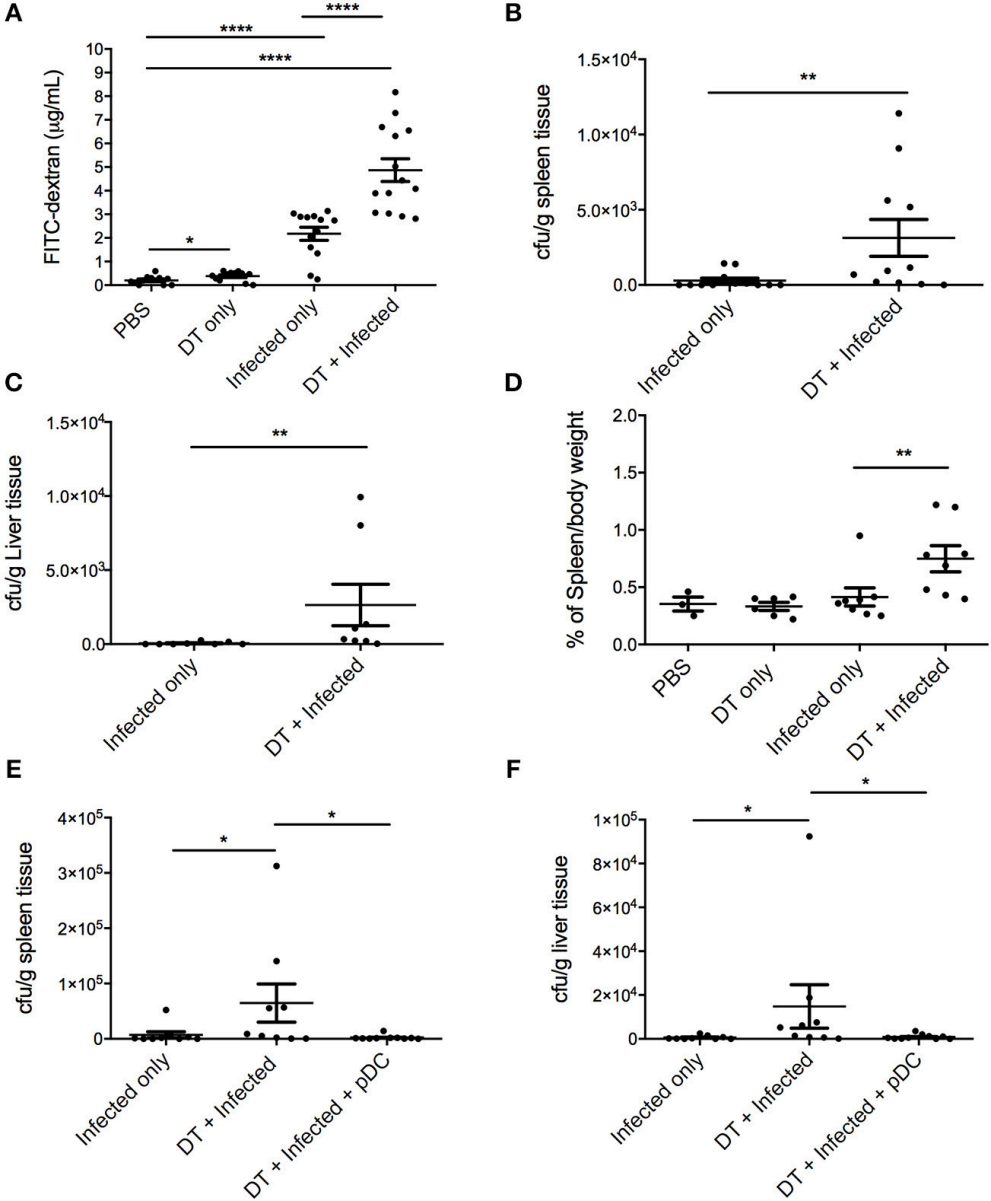
pDC deficiency leads to increased gastrointestinal permeability and systemic spread of *C. rodentium*. BDCA-DTR mice treated as indicated were analyzed 10 days after infection. **(A)** Levels of FITC in serum of mice orally gavaged with FITC-dextran 4 h earlier. CFU of *C. rodentium* in **(B)** spleen and **(C)** liver. **(D)** Spleen weight of pDC-depleted or non-depleted mice at 10 days after *C. rodentium* infection. CFU of *C. rodentium* in **(E)** spleen and **(F)** liver after pDC reconstitution. Each symbol is data of one mouse. Mann-Whitney *U*-test, ^*^P < 0.05, ^**^P < 0.01, ^****^P < 0.0001.

The extent of intestinal permeability was determined at day 10 after infection by assessing leakage of orally administered FITC-labeled dextran into the systemic circulation (Figure 4A). Both pDC depletion alone (“DT only”) and *C. rodentium* infection alone (“Infected only”) resulted in significantly increased intestinal permeability relative to PBS treated mice. However, significantly higher levels of FITC-dextran were found in the serum of the “DT + Infected” mice compared to other groups, including “Infected only,” indicating increased permeability and decreased intestinal integrity following pDC depletion.

A breakdown of the gut mucosal barrier may lead to the escape of gut flora or *C. rodentium* into surrounding tissues and the circulation. To assess this, homogenates of liver and spleen were taken 10 days after infection and cultured for *C. rodentium* (Figures 4B,C). In the “DT + infected” group, 10 of 11 mice had detectable *C. rodentium* in spleen and 8 of 8 mice had detectable *C. rodentium* in liver compared to 5 of 12 in spleens and 3 of 8 in livers of “Infected only” mice. Furthermore, average bacterial loads in the spleens and livers of the “DT + Infected” mice were significantly greater than “Infected only” mice. The spleens from pDC depleted and infected mice were also enlarged in the “DT + Infected” mice compared to mice in all other groups, arguing for a level of bacteria-induced systemic inflammation (Figure 4D). Additionally, unlike the “DT + Infected” group, mice reconstituted with pDC had negligible dissemination of *C. rodentium* to the spleen and liver (Figures 4E,F).

**Figure 5:**
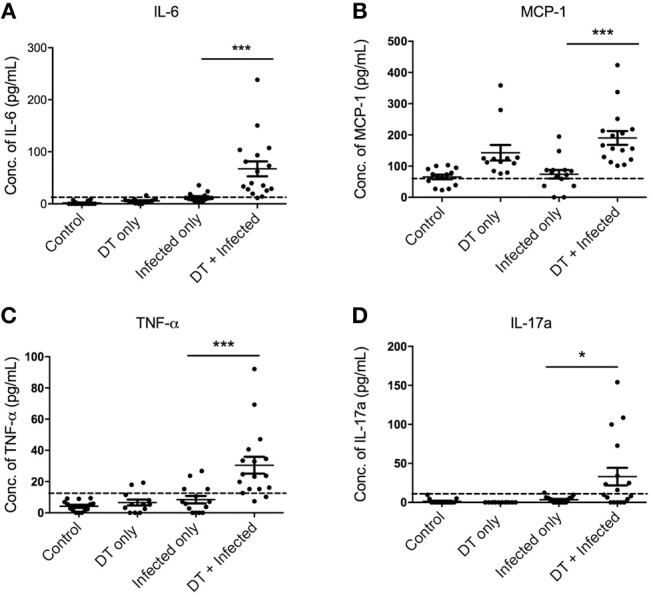
pDC depletion leads to increased inflammatory cytokine levels in serum of *C. rodentium-*infected mice **(A–D)**. BDCA-DTR mice were treated as indicated and serum analyzed for the indicated cytokines 10 days after infection. Data expressed as mean ± s.e.m. Each symbol is data of one mouse. Mann-Whitney *U*-test, ^*^P < 0.05, ^***^P < 0.005.

To gain a better understanding of the inflammatory milieu and how it is affected by pDC depletion during *C. rodentium* infection, we investigated levels of cytokines in the serum using a cytometric bead array assay 10 days after infection. Significantly higher levels of IL-6, MCP1, TNF, and IL-17A were found in “DT + Infected” mice compared to “Infected only” mice (Figures 5A–D). Levels of IFN-γ, IL-10, IL-12, IL-1α, and IL-2 were not significantly different.

Together, these data suggest that colonic epithelial integrity is compromised by pDC depletion allowing *C. rodentium* to enter the systemic circulation and that this in turn led to systemic inflammation.

### Depletion of pDC Leads to Exacerbated Systemic Inflammation Independent of Type I Interferon Signaling

**Figure 6:**
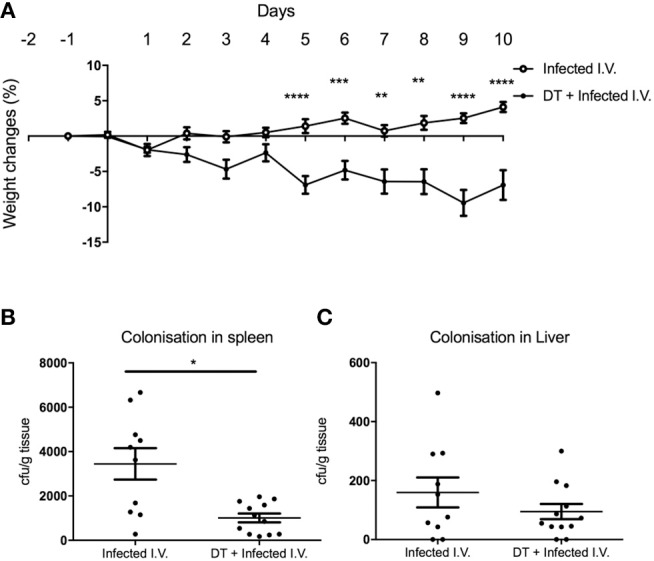
pDC regulate the response to systemic *C. rodentium* infection. BDCA2-DTR mice treated with PBS (“Infected I.V.”) or DT (“DT + Infected I.V.”) on day−1 and infected intravenously with *C. rodentium* on days 0, 2, 4, 6, and 8. **(A)** Weight change. CFU of *C. rodentium* in **(B)** spleen and **(C)** liver 10 days after infection. Data expressed as mean ± s.e.m., *n* = 5 per group in three independent experiments. Mann-Whitney *U*-test, ^*^P < 0.05, ^**^P < 0.01, ^***^P < 0.005, ^****^P < 0.0001.

Although there was a significant difference in weight between “Infected only” and “DT + Infected” mice from day 4 (Figure 2A), there was no difference in fecal bacterial load at day 5 (Figure 2B). This led us to speculate that the phenotype of weight loss was not solely due to increased bacterial load, but a result of dysregulation of the systemic immune response. To test this, we challenged PBS and DT treated BDCA2-DTR mice with *C. rodentium* via the intravenous route (Figure 6). Mice were given i.v. doses of bacteria on day 0, 2, 4, 6, and 8 to mimic the continuous shedding of *C. rodentium* from the gut. “DT + Infected I.V.” mice began to lose weight on day 2 after infection and had lost an average of 8% of their body weight by day 10. In contrast, mice infected by the i.v. route but not depleted of pDC (“Infected I.V.”) gained an average of 4% of their body weight at this time point (Figure 6A). These findings were not explained by differences in bacterial load as the CFU of *C. rodentium* was in fact slightly lower in pDC-depleted vs. non-depleted mice in the spleen (Figure 6B) and was not significantly different between both groups in the liver (Figure 6C). These data suggested that pDCs play a role in regulating the inflammatory response to *C. rodentium* that have disseminated systemically.

**Figure 7:**
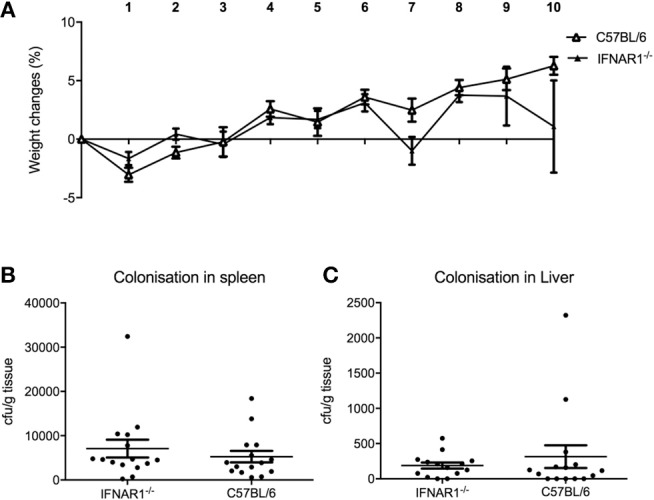
Type I IFN does not influence protection against systemic *C. rodentium* infection. WT and IFNAR1-deficient mice were infected with *C. rodentium* i.v. on days 0, 2, 4, 6, and 8. **(A)** Weight change. CFU of *C. rodentium* in **(B)** spleen, and **(C)** liver 10 days after infection. Data expressed as mean ± s.e.m. *n* = 5 per group in three independent experiments.

Since pDC are the major source of type I IFN, we hypothesized that the mechanism for pDC-mediated protection against systemic inflammation may be dependent on type I IFN. To test this hypothesis, we infected type I interferon receptor-deficient (IFNAR1^−/−^) mice and C57BL/6 wild type (WT) mice with *C. rodentium* via the intravenous route and oral gavage. In contrast to pDC-depleted mice, IFNAR1^−/−^ mice infected i.v. with *C. rodentium* did not lose weight (Figure 7A) during infection and there was no dissemination of bacteria into spleen or liver (Figures 7B,C). In addition, IFNAR1^−/−^ mice showed no weight loss or difference in fecal bacterial load following oral infection (Supplementary Figure 4). These results suggested that type I IFN signaling was not involved in pDC-mediated protection against systemic inflammation after *C. rodentium* infection.

## Discussion

Infection with *C. rodentium* induces many pathological changes in the gut including colonic inflammation, hyperplasia, and diarrhea which also occur during IBD. *C. rodentium* is also commonly used to investigate the host immune response which may provide therapeutic insights for treating IBD. Multiple studies have shown that innate immune cells (e.g., NK cells, macrophages, cDC) (20–22), innate lymphoid cells (11), adaptive T cells (23, 24), and IgG antibody production (25) all contribute to protection or eradication of *C. rodentium* infection. However, the importance of pDC in protection against *C. rodentium* has not been described.

Our results suggested that mice lacking pDC are more susceptible to *C. rodentium* induced gastrointestinal inflammation. pDC depletion in mice led to higher bacterial burden in the gut which probably resulted in increased immune cell infiltration and increased levels of mRNAs encoding inflammatory cytokines in the colon due to the greater degree of inflammatory stimulus. pDC-depleted mice suffered severe weight loss and morbidity during the course of infection, however we suggest the weight loss and morbidity observed in the pDC depleted and infected (“DT + infected”) mice was not simply a direct consequence of the increased bacterial load observed in these mice. The reason for this is because significant weight loss in “DT + Infected” mice was already evident at day 4 after infection, whereas differences in bacterial load were not evident until day 7. Moreover, non-depleted, “Infected only” mice at day 9 had similar bacterial loads to “DT + Infected” mice at day 7, yet at day 9 “Infected only” mice with a normal pDC compartment did not exhibit a dramatic weight loss. Therefore, these observations suggest that the disease phenotype in the pDC-depleted mice was not due to increased bacterial load in the gut and was at least partly a result of a dysregulated immune response to bacteria that escape the gut, as discussed in more detail below.

Histological observation of the gut revealed increased inflammation and enterocyte hyperplasia and abnormal crypt architecture in pDC-depleted mice infected with *C. rodentium*. The mucosal barrier in the gut of “DT + Infected” mice was compromised, as evidenced by increased permeability to FITC-dextran and higher numbers of bacteria in spleen and liver, compared to “Infected only” mice or uninfected mice depleted of pDC. Importantly, there were also signs of increased systemic inflammation in pDC depleted and infected mice including enlarged spleens and higher levels of the cytokines IL-6, MCP1, TNF, and IL-17 in serum. These cytokines were not elevated in serum of “Infected only” mice. It is unlikely that the source of these cytokines was leakage from the colon as mRNAs encoding IFNγ, IL1β, and IL-22 were elevated in the colon of “DT + infected” mice relative to “Infected only” mice and mRNA levels in colon were not different between “Infected only” and “DT + Infected” for TNF and IL-17A. Cytokines found in serum more likely resulted from *C. rodentium*, and/or other gut microflora, leaking from the gut, and stimulating a qualitatively different cytokine response in the “systemic” immune system to that elicited in the gut mucosal immune system.

The systemic dissemination of *C. rodentium* in “DT + Infected” mice was likely due to increased gut barrier permeability. We observed a small but significant increase in intestinal permeability following pDC depletion alone which raised the possibility that pDC may directly contribute to intestinal integrity even in steady state conditions. There is a growing appreciation that immune cells not only combat infections but can also play roles in maintaining tissue integrity in the steady state (26, 27).

*C. rodentium* is known to cause displacement of the microbiome during acute infection. Although we have not performed fecal microbiome studies, since the “Infected only” and “DT + infected” mice were co-housed and we did not observe any differences in fecal consistency between the infected groups (despite the higher load of *C. rodentium* in DT treated mice), we do not believe the phenotype arises from differences in the microbiome over the acute phase of infection.

It is currently unclear whether the increase in systemic inflammation was a direct consequence of *C. rodentium* dissemination or other microflora in the gut. Future work could include identifying which bacterial species are leaking from the gut apart from *C. rodentium* by performing 16S sequencing in spleen and liver (28). However, we did demonstrate that pDC-depleted mice infected intravenously with *C. rodentium* exhibited greater weight loss than systemically infected mice with an intact pDC compartment despite the pDC-depleted mice having a lower bacterial load. This result suggested that pDC may regulate the level of systemic inflammation caused by disseminated *C. rodentium*. This finding is in line with recent demonstrations of pDC with regulatory function rather than solely producers of type I IFNs (29, 30).

Recently, Arimura et al. examined the role of pDC in the development of acute colitis by studying pDC-depleted mice treated with the irritant dextran sodium sulfate (DSS) (31). In contrast to the data shown here, pDC-depleted mice developed *less* severe colitis than wild type mice with lower levels of weight loss, colonic inflammation, and production of inflammatory cytokines. The reason for the different results of Arimura et al. to that shown here is likely due to the different modes of induction of disease and the differential involvement of innate and adaptive immune responses. DSS is thought to cause colitis by damaging gut epithelia and allowing gut commensal organisms to leak into the lamina propria inducing an innate inflammatory response that is independent of T cells (32). Our work suggested that the more severe disease observed in pDC-depleted mice infected with *C. rodentium* may be the result of immune dysregulation in response to bacteria released from the gut. Such an effect may not be evident in DSS treated mice where commensal bacteria do not illicit as strong an immune response. Of relevance, Mizuno et al. suggested that CCR9^+^ pDC can suppress T cell-mediated inflammation of the small intestine, a finding similar to our result in the colon (33). Future work in this system could include a more detailed analysis of the systemic adaptive T and B cell response to *C. rodentium* and other gut microflora.

An obvious potential mediator of the effects of pDC on systemic inflammation is type I interferon. Here, mice deficient for type I IFN signaling (IFNAR1^−/−^) were infected with *C. rodentium* intravenously. Since IFNAR1^−/−^ mice did not lose weight during the course of infection, this suggested that the effect of pDC depletion was not due to lack of type I IFN signaling. Apart from type I IFN, pDC have been shown to produce type III IFN upon viral infection (34, 35). Type III IFN induces similar sets of interferon stimulated genes as type I IFN, suggesting some redundancy in function. Moreover, type III IFN contributes to immune protection against *Listeria monocytogenes* in intestinal epithelial cells which is also the primary site for *C. rodentium* infection (36). Therefore, more work is needed to investigate the role of type III IFN during *C. rodentium* infection and the possible relationship with pDC function during gut infection.

In conclusion, our data indicates that pDC have a protective role in limiting bacterial load in the gut and helping to maintain the intestinal barrier. As *C. rodentium* is closely related to the attaching and effacing human pathogens EPEC/EHEC, our work suggests that pDC may also play a role in protection from these organisms. More generally, a role for pDC in maintenance of the gut mucosal barrier and perhaps gut homeostasis would mean these cells may be important in protection from many human gut pathogens. pDC also appeared to regulate the systemic inflammatory response to pathogens released from the gut. Although pDC-depletion was found to ameliorate DSS-induced colitis, our results and those of Mizuno et al. showed that a deficit in pDC led to exacerbation of disease in infection and T cell-dependent models of IBD. Hence, caution is warranted when considering the manipulation of pDC activity in gut pathologies (33).

## Author Contributions

EH and IvD: conceptualization; TR, AB, EH, IvD, and KF: methodology; TR, IvD, and KF: formal analysis; TR and KF: investigation; TR, IvD, and KF: writing–original draft; TR, AB, EH, IvD, and KF: writing–review and editing; EH, IvD, and KF: supervision; AB, EH, IvD, and KF: project administration; EH and IvD: funding acquisition.

### Conflict of Interest Statement

The authors declare that the research was conducted in the absence of any commercial or financial relationships that could be construed as a potential conflict of interest.
