# Supplementary material for: Plasmacytoid Dendritic Cells Provide Protection Against Bacterial-Induced Colitis
Source: Front Immunol. 2019 Apr 9;10:608. doi: 10.3389/fimmu.2019.00608 (PMC6465541; doi:10.3389/fimmu.2019.00608)
Supplement: Supplementary file 1 [file Data_Sheet_1.pdf]

frontiers

Supplementary Material

Plasmacytoid Dendritic Cells Provide Protection Against Bacterial-Induced Colitis

Tania Rahman[1,2,3], Andrew S Brown[2], Elizabeth L Hartland[1,] Ian R van Driel[2*] and Ka Yee Fung[1,3]

1. Department of Microbiology and Immunology, University of Melbourne at the Peter Doherty Institute for Infection and Immunity, Melbourne, Victoria, Australia

2. Department of Biochemistry and Molecular Biology, Bio21 Molecular Science and Biotechnology Institute, University of Melbourne, Melbourne, Victoria, Australia,

3. TR and KYF made similar contributions to this work.

4.

TR current address: Department of Biochemistry and Molecular Biology, University of Dhaka, Bangladesh

ASB current address: Laboratory of Immunoregulation and Mucosal Immunology,VIB-UGent Center for Inflammation Research, Technologiepark-Zwijnaarde 71, 9052 Ghent, Belgium

ELH current address: Centre for Innate Immunity and Infectious Diseases, Hudson Institute of Medical Research, 27-31 Wright Street, Clayton, VIC 3168, Australia and Department of Molecular and Translational Science, Monash University, Clayton 3168, Australia

KYF current address: Personalized Oncology Division, Walter and Eliza Hall Institute of Medical Research, 1G Royal Parade, Parkville, VIC 3052, Australia

*** Correspondence:** Prof Ian R van Driel: i.vandriel@unimelb.edu.au

1 Supplementary Table

Gene name	Sequence
18S F’	GTAACCCGTTGAACCCCATT
18S R’	CCATCCAATCGGTAGTAGCG
Ifnγ F’	GCAACAGCAAGGCGAAAAAG
Ifnγ R’	CTCATTGAATGCTTGGCGCT
Il1β F’	TCTGGGATCCTCTCCAGCCAAG
Il1β R’	TCAGGACAGCCCAGGTCAAAGG
Il22 F’	TTGAGGTGTCCAACTTCCAGCA
Il22 R’	AGCCGGACATCTGTGTTGTTA
Il17a F’	ACCGCAATGAAGACCCTGAT
Il17a R’	TCCCTCCGCATTGACACA
Tnfα F’	CAAATTCGAGTGACAAGCCTG
Tnfα R’	GAGATCCATGCCGTTGGC

Supplementary Table 1. Primers used for qPCR for the genes indicated

1.1 Supplementary Figures

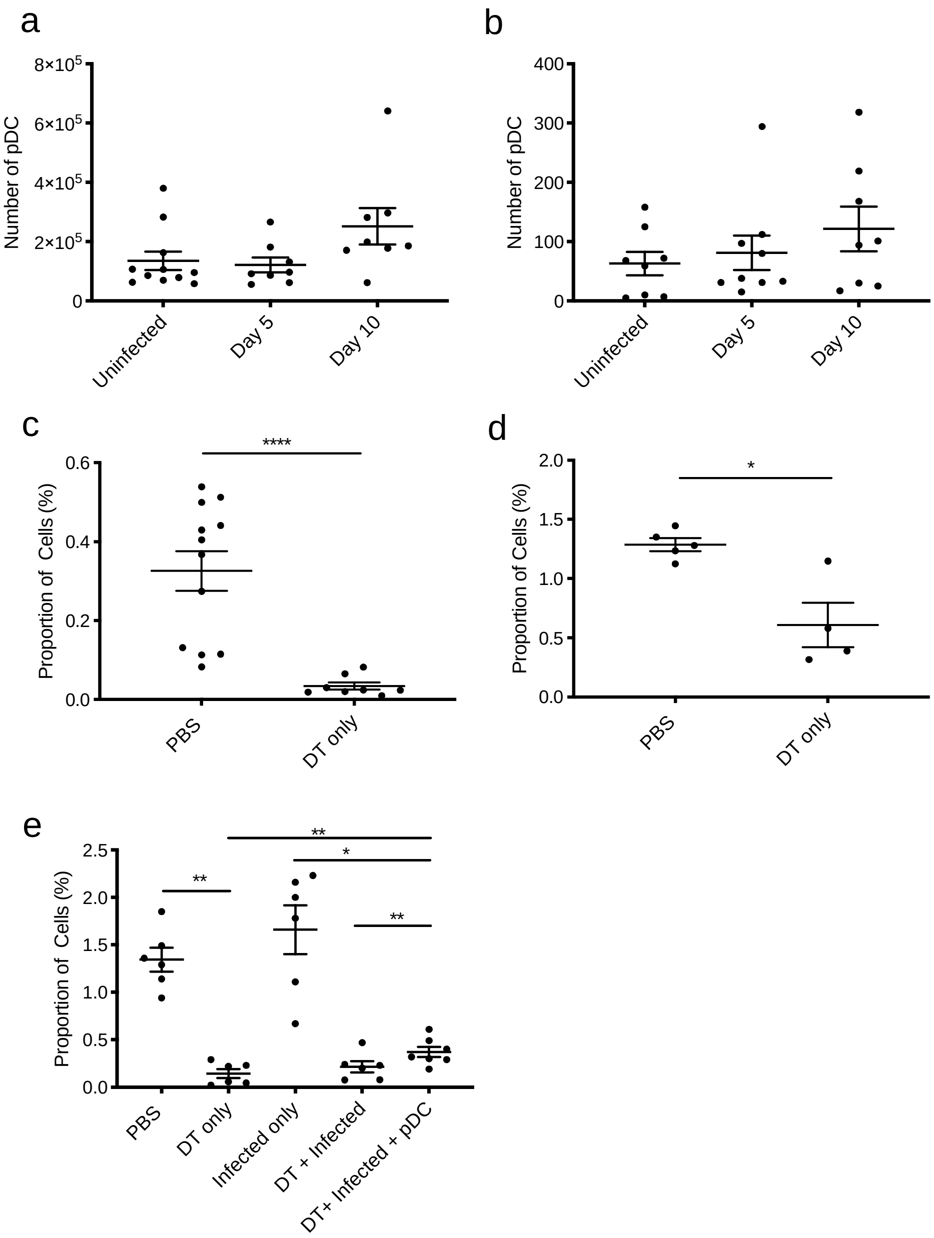

Supplementary Figure 1. pDC depletion and reconstitution. Number of pDC in (a) spleen and (b) colon on day 10 after infection. Proportion of $CD45^+$ cells identified as pDC in (c) spleen and (d) colon of BDCA2-DTR mice on day 10 after infection in mice treated with PBS or DT. (e) Proportion of $CD45^+$ cells identified as pDC in 'reconstitution experiment shown in Figure 2c and 2d on day 10 after infection. Each symbol is data for one mouse. Mann-Whitney T test, * *P<0.05, ** P<0.01, ****P<0.001.*

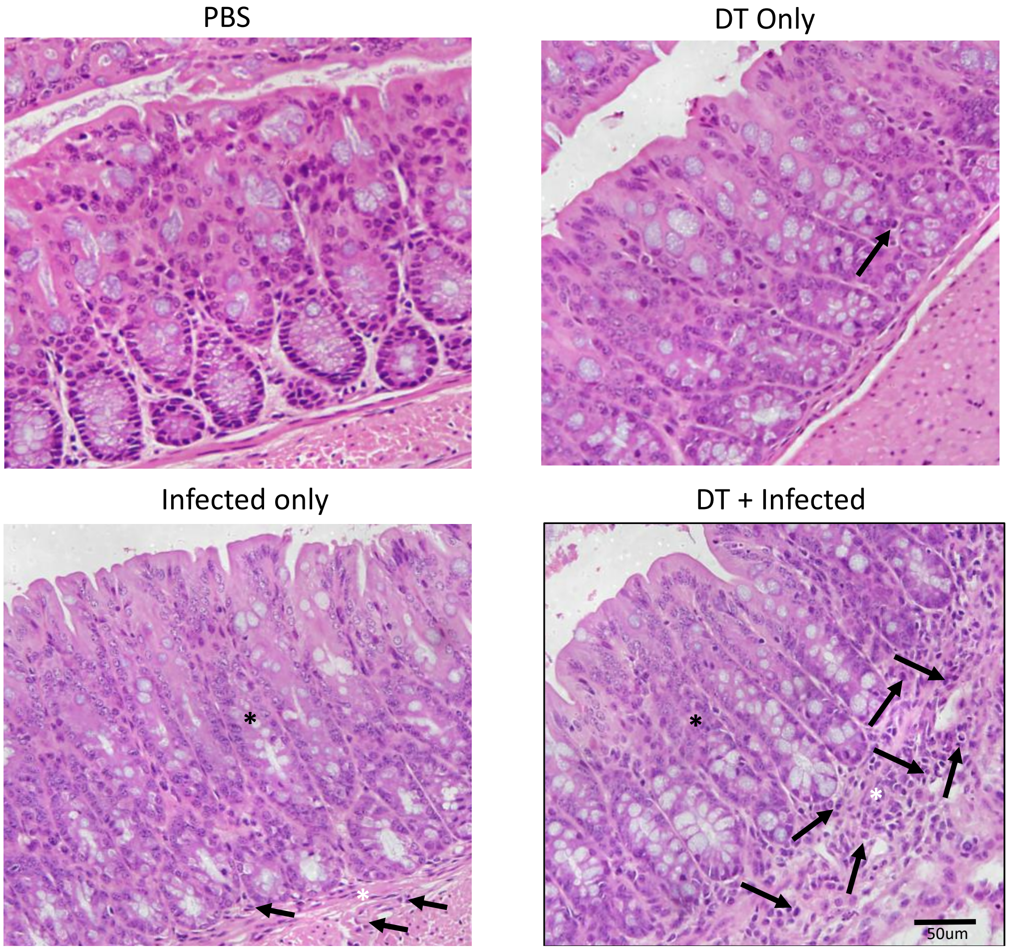

Supplementary Figure 2. Histopathology in pDC depleted mice after C. rodentium infection. Representative haemotoxylin and eosin stained sections of mouse colons from the experimental groups indicated. Mice were killed and tissue taken at day 10 after infection. Black asterisks indicate elongated crypts. White asterisks indicate submucosal edema. Black arrows indicate inflammatory infiltrates.

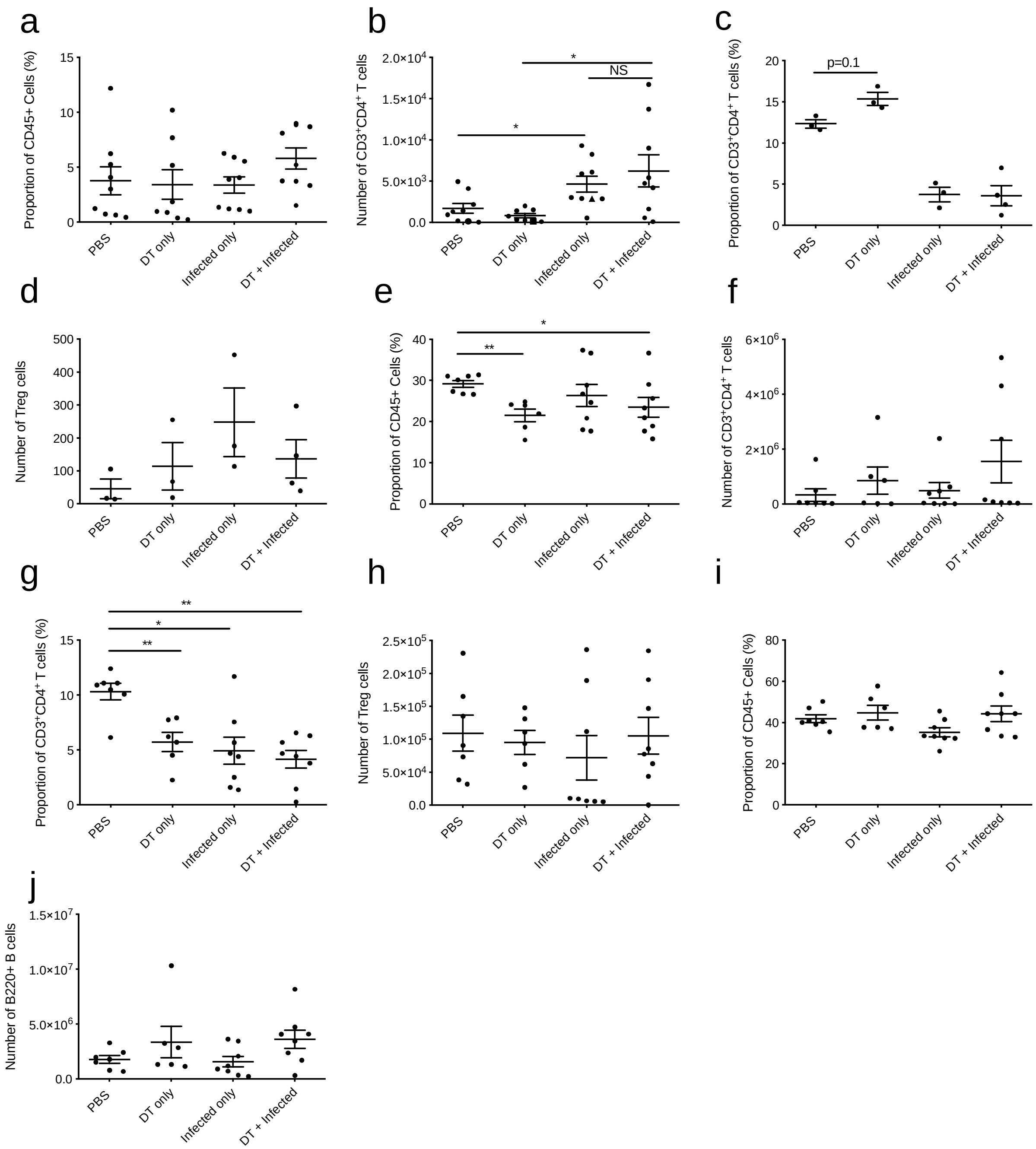

Supplementary Figure 3. Analysis of CD4+ T cells, Treg cells and B cells in *C. rodentium* and pDC depleted mice. Samples from mice analysed 10 days after infection are shown. The proportions and number of $CD4^+$ T cells in colon (a, b). The proportions and number of $Foxp3^+$ Treg cells in colon (c, d). The following samples were from mesenteric lymph nodes. Proportion and number of $CD4^+$ T cells (e, f). Proportion and number of $Foxp3^+$ Treg cells (g, h). Proportion and number of $B220^+$ B cells (i, j) Each symbol is data for one mouse. Mann-Whitney T test, * $P<0.05$, ** $P<0.01$.

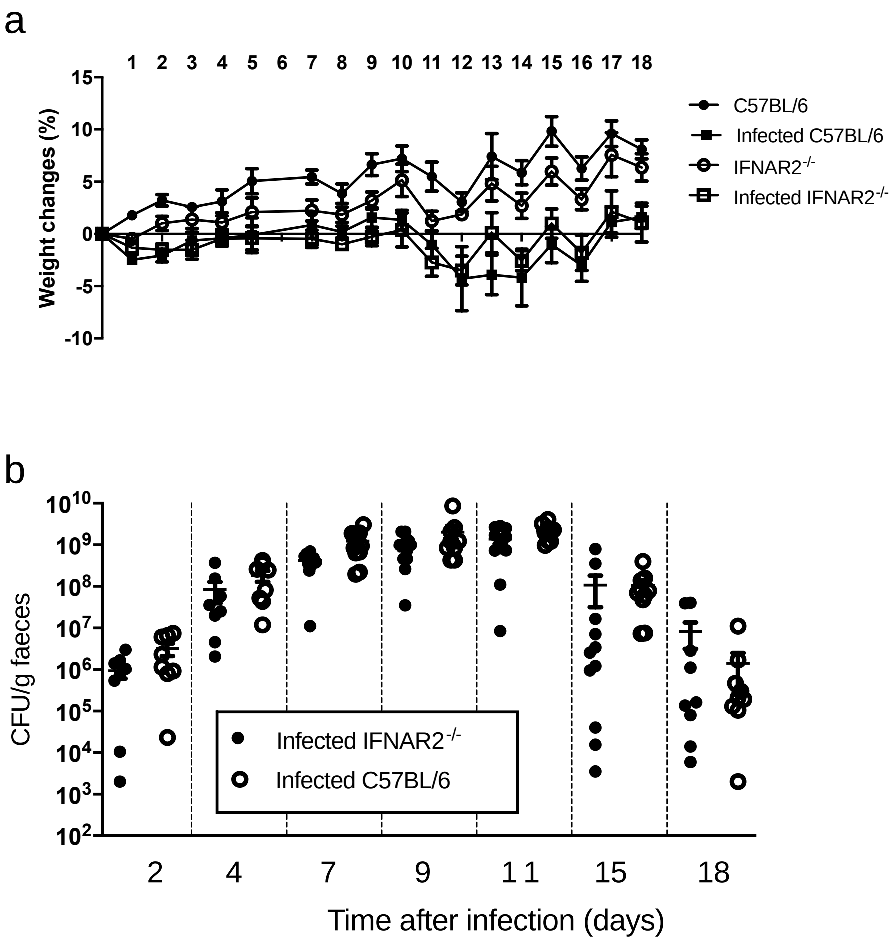

Supplementary Figure 4. Mice deficient in type 1 interferon signalling show similar weight loss and bacterial loads after *C. rodentium* infection. Mice deficient in IFNAR2 or WT C57BL/6 mice were infected orally with *C. rodentium* on day 0. (**a**) Weight change of mice during the course of infection. Data expressed as mean ± s.e.m. (**b**) *C. rodentium* CFU in faecal pellets. Each symbol is data for one mouse.
